# Unpicking Causal Relationships Between Grip Strength and Cardiorespiratory Fitness: A Bidirectional Mendelian Randomization Study

**DOI:** 10.1111/sms.14775

**Published:** 2024-12-06

**Authors:** Tom Norris, Rachel Cooper, Victoria Garfield, Mark Hamer, Snehal M Pinto Pereira

**Affiliations:** ^1^ Division of Surgery and Interventional Science, Faculty of Medical Sciences University College London London UK; ^2^ AGE Research Group, Translational and Clinical Research Institute, Faculty of Medical Sciences Newcastle University Newcastle upon Tyne UK; ^3^ NIHR Newcastle Biomedical Research Centre, Newcastle Upon Tyne NHS Foundation Trust, Cumbria, Northumberland Tyne and Wear NHS Foundation Trust and Newcastle University Newcastle upon Tyne UK; ^4^ Unit for Lifelong Health and Ageing at UCL, Institute of Cardiovascular Science University College London London UK; ^5^ Department of Pharmacology and Therapeutics University of Liverpool Liverpool UK

**Keywords:** cardiorespiratory fitness, grip strength, Mendelian randomization, UK Biobank

## Abstract

Understanding the dominant direction of association between cardiorespiratory fitness (CRF) and grip strength could help refine physical activity recommendations. We performed a Mendelian Randomization (MR) analysis to elucidate the bidirectional relationship between CRF and grip strength (GS). Using an inverse‐variance weighted (IVW) MR framework, we estimated the strength of the GS (exposure)‐CRF (outcome) association using genome‐wide association summary data. When examining the CRF (exposure)‐GS (outcome) association, the CRF genetic instrument was related to individual‐level GS phenotypic data in 367 693 UK Biobank participants. Several sensitivity analyses were performed (e.g., MR‐Egger, MR‐weighted median estimator and MR‐PRESSO) and both measures were scaled by body weight (w). In the direction GS‐to‐CRF, a 1‐unit increase in GSw (i.e., GS/weight) was associated with 1.70 mL/kg/min (95% confidence interval (CI): 1.14,2.27) higher CRFw (IVW model). This finding persisted across most sensitivity analyses. In the reverse direction, there was no evidence supporting an effect of CRFw on GSw, e.g., a 1‐unit increase in CRFw led to a 0.00 kg/kg (95% CI: −0.01,0.02) higher GSw (IVW model). Our finding of a dominant direction of association from greater GS to higher CRF is relevant when considering how to promote physical activity guidelines. For example, placing too much emphasis on improving/maintaining CRF is unlikely to result in maximum benefits for other fundamental components of physical fitness, particularly muscle strength.

## 1 Introduction

Cardiorespiratory fitness (CRF) and muscle strength are essential for health. Low levels of CRF are strongly associated with greater risk of premature mortality, incident chronic conditions, and adverse cardiovascular events [1]. Similarly, low muscle strength is associated with all‐cause and cause‐specific mortality [2, 3], hypertension and the metabolic syndrome [3]. Both CRF and muscle strength decline as age increases from midlife [4, 5]; although aerobic and strength‐building activities can slow down and/or delay declines in CRF and muscle strength respectively. These traits develop and change over the life‐course [6, 7] and a growing evidence base highlights the importance of developing and maintaining high levels of CRF and muscle strength throughout life [8, 9].

Whilst CRF and muscle strength are distinct phenotypes conferring independent health benefits [10], they are likely to be inter‐related. For example, a meta‐analysis of 39 trials in participants, aged > 60 years, found that those assigned to resistance/strength‐based interventions increased their CRF [11]. Conversely, a systematic review of trial evidence from older adults did not support an effect of aerobic exercise interventions on strength outcomes [12]. However, most previous studies have focused on older populations and, to our knowledge, have not examined the potential bi‐directional relationships between CRF and muscle strength simultaneously. Public health guidelines recommend adults engage in both aerobic and strength‐building activities [13] to improve/maintain overall health and both CRF and muscle strength, respectively. However, surveillance indicates that while 60%‐to‐70% of adults meet aerobic exercise guidelines [14], only 17% meet muscle strengthening guidelines [15]. This substantial attainment gap highlights the need for increased emphasis on muscle strength (and how to improve it) in public health initiatives. This is particularly relevant in our current aging population as greater muscle strength has been shown to reduce the risk of falls, fractures, and physical disability [16]. Therefore, understanding whether there is a prevailing direction of association between CRF and strength, over a broad age‐range, will help determine whether performing activities related mostly to one aspect of physical fitness (e.g., strength) may confer additional benefits to another aspect (e.g., CRF). This information will benefit population health for multiple reasons. It could raise awareness that focusing on one type of activity, may (or may not), be sufficient to improve/maintain both physical fitness phenotypes. Additionally, this knowledge could help further refine and personalize future physical activity recommendations.

Thus, to address current knowledge gaps, we aim to identify whether, on average over a lifetime, a prevailing direction of causality exists between CRF and muscle strength. We do this using genetic instruments for CRF and muscle strength obtained from published genome‐wide association studies (GWAS) [17, 18] in combination with phenotypic data from participants in the UK Biobank study (UKB) [19]. Specifically, the instruments used were for estimated V0_2_‐max (from a submaximal test) and grip strength (GS; a commonly used proxy for overall muscle strength); both were scaled for body weight.

## 2 Methods

### 2.1 Study Design

**FIGURE 1:**
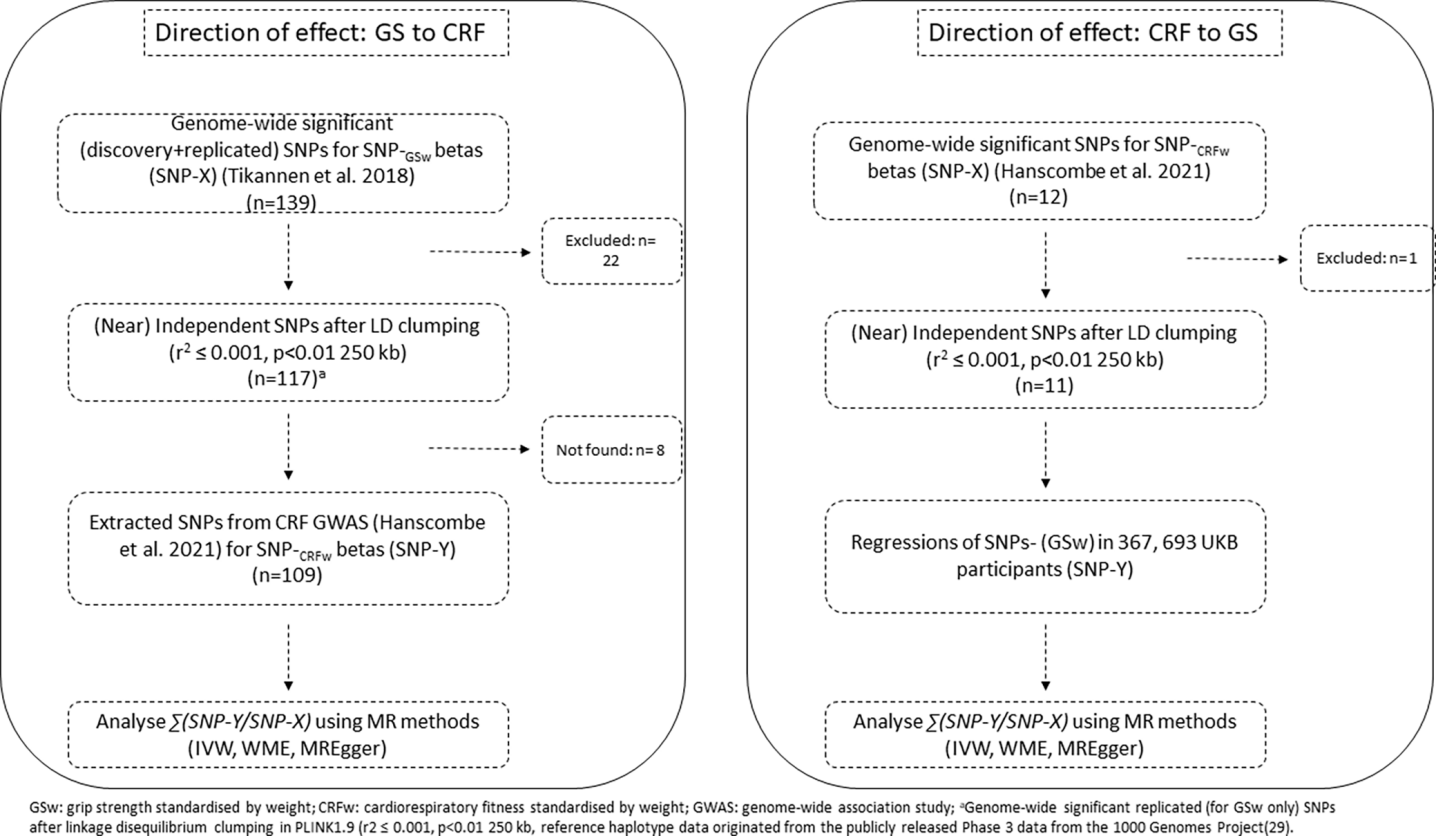
Study design illustrating the combination of summary‐level and individual‐level data.

We employed a bidirectional Mendelian randomization (MR) approach, whereby summary‐level genome‐wide association data were combined with individual‐level phenotypic data (Figure 1, Table 1). Specifically, in the direction of GS (exposure) to CRF (outcome), we used summary‐level data for both GS and CRF, obtained from their respective GWAS [17, 18]. In the direction of CRF (exposure) to GS (outcome), we combined summary‐level data for CRF with individual‐level genetic & phenotypic data obtained from 367,693 UKB participants. Below we describe the phenotypic measures used from UKB and our adopted MR approaches; for more details see ‘Supplementary methods’ (Data S1).

**TABLE 1:** Summary of genome‐wide association studies (GWAS) examined.

Direction of effect	GWAS used for instrument curation	Instrument details	Notes
Grip strength ➔ cardiorespiratory fitness	GS_w_: Tikkanen E et al. *Scientific Reports* 2018;8(1):6451.	Genome‐wide significant (discovery + replicated) SNPs for SNP‐GS_w_ betas (see Figure 1)	Two‐sample MR with overlapping samples. *NB: corresponding SNPs extracted from CRF*_*w*_ *GWAS (Hanscombe KB et al. Genome Medicine 2021;13(1):1–19)*
Cardiorespiratory fitness ➔ grip strength	CRF_w_: Hanscombe KB et al. *Genome Medicine* 2021;13(1):1–19	Genome‐wide significant SNPs for SNP‐CRF_w_ betas (see Figure 1)	*Pseudo* two‐sample MR (SNP‐CRF_w_ from GWAS; SNP‐GS_w_ from individual level data in UKB participants)

*Note:* GS_w_: grip strength standardized by weight; CRF_w_: cardiorespiratory fitness standardized by weight.

### 2.2 Phenotypic Measures in UK Biobank: Grip Strength and Weight Measurement

UKB is a large prospective cohort of individuals aged 40–69 years at recruitment (2006–2010) from across the United Kingdom [19]. The sample examined here included 367,693 European ancestry participants with available data on genotypes and, at baseline, valid weight and GS measures (details in Supplementary methods [Data S1]). Grip strength was assessed using a Jamar J00105 hydraulic hand dynamometer. Participants sat upright in a chair with their forearms on armrests. They were asked to squeeze the dynamometer's handle as hard as they could with their right hand for about 3 s. The grip strength measurement was then repeated using the same protocol for the left hand [20]. We examined the maximum recorded value (greater than 0) from either hand. Weight (kg) was measured using a Tanita BC‐418 MA body composition analyzer. To be consistent with the scaling metric of genetic instruments for GS and CRF [17, 18], we divided GS by weight to obtain relative grip strength, i.e., grip strength per kg of body weight (GS_w_).

### 2.3 GS to CRF Instrument Curation: Obtaining SNP‐X[GS] and SNP‐Y[CRF] Coefficients

We used summary level data from a GWAS on GS/weight [17]. Hence, our instrument is for GS standardized by weight (GS_w_). This instrument (SNP‐X_GSw_) comprised 109 near‐independent SNPs which achieved genome‐wide significance (*p* < 5 x 10^−8^); details in Supplementary methods (Data S1), Figure 1 and Table 1. These 109 SNPs were then extracted from the CRF GWAS [18] (SNP‐Y_CRFw_), which was also standardized by weight (details below). Where necessary, SNP‐X_GSw_ beta coefficients were multiplied by −1 to ensure all betas represented an increase in GSw. Next, allele harmonization was done, ensuring alignment of SNP‐Y_CRFw_ alleles to SNP‐X_GSw_ associations. The 109‐SNP instrument for SNP‐X_GSw_ had an F‐statistic of 34.38, explaining 1.01% of variability in GS_w_.

### 2.4 CRF to GS Instrument Curation: Obtaining SNP‐X[CRF] and SNP‐Y[GS] Coefficients

We leveraged data from a CRF (scaled for weight) GWAS for estimated V0_2_‐max (from submaximal testing) [18]. We curated an instrument (SNP‐X_CRFw_) comprising 11 near‐independent SNPs which achieved genome‐wide significance; details in Supplementary methods (Data S1), Figure 1 and Table 1. F‐statistic and variance explained details were not reported in the original GWAS [18]. Additionally, as CRF phenotypic data was not available in UKB, it was not possible to calculate the CRF instrument's F‐statistic or proportion of CRF variance explained using individual‐level data. Thus, we calculated an approximate F‐statistic using the ‘t‐statistic’ method [21]; this value was 37.72. To obtain SNP‐Y_GSw_ associations, these 11 SNPs were regressed against GS_w_ in 367 693 UKB participants (further details in Supplementary methods [Data S1]).

### 2.5 Statistical Analysis

The described analyses were performed initially with GSw instruments as the exposure and CRFw as the outcome and then vice‐versa.

We applied the inverse‐variance weighted (IVW) method as our main MR model. This method estimates the causal effect of an exposure on an outcome by averaging the genetic instruments' ratio of instrument–outcome (SNP‐Y) to instrument–exposure (SNP‐X) association estimates using a multiplicative random effects meta‐analysis model. We report the I^2^ statistic to quantify the extent of heterogeneity between SNP‐specific causal estimates. For the only individual level genetic association estimated (i.e., SNP‐Y_GSw_) via linear regression in PLINK 2.0, we adjusted for 10 genetic principal components. We performed three additional MR analyses: MR‐Egger, weighted median estimator (WME), and MR‐PRESSO. MR‐Egger yields an intercept term which indicates the presence of unbalanced horizontal pleiotropy (i.e., if genetic instruments are associated with the outcome via pathways other than the exposure); WME provides more robust estimates when up to 50% of the genetic variants are invalid and MR‐PRESSO identifies and corrects for potential outliers (details in Supplementary methods [Data S1]).

### 2.6 Sensitivity Analyses

For MR analyses to be valid, three key assumptions must be met: (i) genetic variants should be robustly associated with the exposure; (ii) genetic variants should be independent of confounding factors of the relationship in question; and (iii) the association between the genetic variants for the exposure and the outcome must only operate via the exposure under study (i.e., no unbalanced horizontal pleiotropy). We explored the validity of these assumptions by testing associations between SNPs and potential confounders (sex, height, weight, depression, arthritis, asthma, area‐level deprivation, smoking status, physical activity, age, and alcohol intake) in UKB. We applied a Benjamini‐Hochberg false discovery rate of 0.05 to account for multiple testing. Where associations were observed, MR analyses were re‐run excluding potentially invalid SNPs. Additionally, when the MR‐Egger intercept indicated pleiotropy (*p* < 0.05), we undertook further analyses: outlying SNPs and those with a large influence on the estimates were identified by (i) funnel plots and (ii) Cook's Distance [22]. We re‐ran analyses removing the identified SNPs (details in Supplementary methods [Data S1]).

We used Stata17, R4.3.0, and PLINK1.9/2.0 for data processing and statistical analyses. MR analyses were performed using the *mrrobust* (Stata) [23] and MR‐PRESSO (R) [24] packages. This manuscript is reported according to current guidelines (Supplementary Table 1) and summary statistics describing SNP‐CRFw and SNP‐GSw associations are presented in Supplementary Table 2.

### 2.7 Ethics Statement

Ethical approval for UKB was given by the National Information Governance Board for Health and Social Care and North‐West Multicentre Research Ethics Committee. The approval covered the analysis of all data in the present study. Participants provided informed consent; the current study is approved by UKB (application number: 71702).

## 3 Results

Mean age of the 367,693 UKB participants included in the CRF_w_‐GS_w_ MR was 56.7 years (standard deviation (SD)): 8.0) (Table 2). 46.3% of the sample were male, 53.7% were female. While absolute maximum GS varied by sex (41.9 kg (SD:8.9) in males vs. 25.1 kg (SD:6.3) in females), relative GS was similar in males (0.5, SD:0.1) and females (0.4, SD:0.1).

**TABLE 2:** Sample characteristics (*n* = 367 693).

Variable	N(%) / Mean (SD)
*Sociodemographic characteristics*	
Sex	
Male	170 223 (46.3)
Female	197 470 (53.7)
Age at recruitment (years)	56.7 (8.0)
Townsend deprivation index^a^, ^b^	−2.4 (−3.8, −0.0)
Current smoker	
No	331 636 (90.2)
Yes	36 057 (9.8)
Alcohol consumption	
Less than daily	288 459 (78.5)
Almost/daily	79 234 (21.5)
Physical activity	
Active (vigorous activity ≥ 4×/wk)	68 140 (18.5)
Inactive (vigorous activity < 4×/wk)	299 553 (81.5)
*Health characteristics*	
Maximum grip strength (*kg*)	
Males	41.9 (8.9)
Females	25.1 (6.3)
Relative grip strength *(kg/body weight in kg*)	0.4 (0.1)
Males	0.5 (0.1)
Females	0.4 (0.1)
Weight (*kg*)^a^	76.6 (66.7, 87.7)
Males	84.6 (76.5, 94.0)
Females	69.2 (61.9, 78.6)
Height (*m*)	1.7 (0.1)
Males	1.8 (0.1)
Females	1.6 (0.1)
Arthritis	
No	333 793 (90.8)
Yes	33 900 (9.2)
Asthma	
No	325 349 (88.5)
Yes	42 344 (11.5)
Depression^c^	
No	347 193 (94.4)
Yes	20 500 (5.6)

^a^ Summarized as median (25^th^, 75^th^ centile).

^b^ A higher index indicates more deprivation.

^c^ Self‐reported at baseline.

### 3.1 GSw to CRFw

Estimates from each of the four MR models (IVW, WME, MR‐Egger, and MR‐PRESSO) indicated an increasing and consistent effect of GSw on CRFw. For example, a 1‐unit increase in GSw was associated with a 1.70 mL/kg/min (95% confidence interval (CI); 1.14, 2.27) higher CRFw in the IVW model (Table 3, Supplementary Figure 1). Many SNPs (102) included in the GSw instrument were associated with examined potential confounders. When these SNPs were removed and analysis re‐run, effects were consistent with those in the main analysis in terms of direction and magnitude, but confidence intervals straddled the null (Supplementary Table 3). For example, the IVW estimate suggested a 1‐unit increase in GSw was associated with a 1.66 mL/kg/min (95% confidence interval (CI): −0.14, 3.47) higher CRFw. While MR‐Egger did not provide evidence of an overall effect of horizontal pleiotropy (MR_Egger_ p‐value_intercept_ = 0.27), funnel plots and Cook's Distance identified seven potentially pleiotropic SNPs (rs115131074, rs117642368, rs13135092, rs17024393, rs17249398, rs181617194, rs76808502). When analysis was re‐run without these SNPs, consistent effects to those reported above were observed. For example, the IVW estimate suggested a 1‐unit increase in GSw was associated with a 1.16 mL/kg/min (95% CI:1.10, 2.22) higher CRFw (Supplementary Table 4). Re‐running analyses after removing all 103 SNPs associated with confounders and/or showing potentially pleiotropic effects resulted in directionally consistent estimates, but with confidence intervals straddling the null (e.g., IVW effect: 1.18 mL/kg/min (95% CI:‐0.83, 3.19) higher CRFw) (Supplementary Table 5). Similarly, MR‐PRESSO provided consistent results. The global test was significant (*p* < 0.001), indicating the presence of some horizontal pleiotropy. However, this only had a small effect on the causal estimate, as indicated by a non‐significant MR‐PRESSO distortion test (*p* = 0.71) and an outlier‐adjusted estimate of 1.63 mL/kg/min (Table 3).

**TABLE 3:** MR estimates of the bidirectional associations^a^ between GSw‐CRFw.

*Difference (95% CI) in CRFw (ml/kg/min) per 1‐unit increase in GSw*	
*Number of SNPs*	109
IVW	1.70 (1.14, 2.27)
I^2^	0.39
WME	1.72 (1.01, 2.44)
MR‐Egger	3.08 (0.57, 5.59)
*p‐*pleiotropy	0.27
MR‐PRESSO	
Outlier corrected causal estimate	1.63 (*p* < 0.001)
Global test	*p* < 0.001
Distortion test	*p* = 0.71
***Difference (95% CI) in GSw (kg/kg) per 1‐unit increase in CRFw***	
*Number of SNPs*	11
IVW	0.00 (−0.01, 0.02)
I^2^	0.81
WME	0.00 (−0.01, 0.01)
MR‐Egger	0.02 (−0.02, 0.06)
*p*‐pleiotropy	0.34
MR‐PRESSO	
Outlier corrected causal estimate	0.00 (*p* = 0.35)
Global test	*p* < 0.001
Distortion test	*p* = 0.77

Abbreviations: IVW: Inverse‐variance‐weighted; WME: weighted median estimator; MR‐Egger: Mendelian randomization Egger regression; MR‐PRESSO: Mendelian Randomization Pleiotropy RESidual Sum and Outlier analysis. ^a^Associations between genetically predicted increases in GSw on CRFw and vice versa.

### 3.2 CRFw to GSw

In the other direction, across all MR models, there was no evidence in support of an effect of CRFw on GSw. For example, a 1‐unit increase in CRFw led to a 0.00 kg/kg (95% CI:‐0.01, 0.02) higher GSw in the IVW model (Table 3, Supplementary Figure 1). Removing SNPs associated with confounders and/or showing potentially pleiotropic effects did not substantively change estimates (Supplementary Tables 3–5). MR‐PRESSO indicated the presence of some horizontal pleiotropy (global test *p* < 0.001), but this had no substantial impact on the causal effect (or lack thereof) of CRFw on GSw (Table 3).

## 4 Discussion

We investigated evidence for causal links between CRF and GS in both directions in large‐scale GWAS and UKB, using several complementary MR approaches and found important differences in terms of the postulated directions of association. In the direction CRF‐GS, all MR estimates were consistent with the null, suggesting, on average over a lifetime, no causal effect of CRF on GS. In the reverse direction, estimates of the effect GS on CRF were directionally consistent across all MR analyses, indicating, on average over a lifetime, higher GS resulted in higher CRF.

Evidence from the literature is sparce regarding the existence and/or direction of association between CRF and GS. However, similar to our findings, in 846 Finnish males aged between 20 and 30 years, Vaara et al. [25] observed no evidence of an association from CRF (i.e., V0_2_‐max, scaled for weight) to absolute GS. More broadly, our findings agree with a previous meta‐analysis of 39 trials in participants aged > 60 years, examining a behavior (i.e., assignment to resistance/strength‐based interventions) and its impact on CRF [11]. They observed that those assigned to resistance/strength‐based interventions increased CRF (assessed by V0_2_‐peak) by, on average, 1.89 mL/kg/min, which broadly aligns (in terms of magnitude) to our findings. Effects of strength‐based training on improved CRF have also been observed in other population groups, e.g., young adults [26] and post‐menopausal women [27], adding further support to our findings of an effect over the lifetime. In the direction CRF to muscle strength, in line with our findings, trial evidence does not support an effect of aerobic exercise interventions on strength outcomes [12]. Despite these consistencies, it is important to note a distinction between our work and most previous literature. Here, we examine the relationship between two phenotypes (CRF and GS), whereas the above quoted studies examine (assignment to) associated behaviors. It is possible, for example, that many aerobic activities that people commonly engage with (e.g., running, football, swimming) improve both CRF and strength via independent mechanisms, despite there being no evidence of a causal association from CRF to muscle strength.

While it is unclear how a person's genetic predisposition to higher GS may increase their CRF, at least three situations warrant consideration. First, the role of horizontal pleiotropy, in which SNPs are associated with CRF via other traits (which are not downstream of strength), needs to be considered, as indicated by the significant MR‐PRESSO global test. For example, several SNPs included in the GSw instrument (e.g., rs10807136, rs10203386, rs11645565, rs117642368, rs62004866, rs1642294, rs34633411) have exhibited relationships with forced expiratory volume [28]. This indicates a potential role of the included SNPs on lung function, which can then plausibly be related to CRF levels. Indeed, SNP rs117642368 was identified as one of the SNPs demonstrating high influence on the GSwꟷCRFw MR estimate, indicating a potentially horizontal pleiotropic effect. Two further SNPs are associated with functioning of cardiac muscle tissue (rs1627854 and rs4952499), which may represent another pleiotropic pathway via which strength and CRF may be associated. Reassuringly, when analyses were re‐run excluding all identified potential pleiotropic SNPs, the effect of GSw on CRFw still persisted (per unit increase in GSw, CRFw was higher by 1.16 mL/kg/min (95% CI:1.10, 2.22), IVW estimate). Moreover, our MR‐Egger and MR‐PRESSO analyses did not indicate a substantial effect of horizontal pleiotropy as evidenced by the concordant outlier‐adjusted estimates, which were similar in magnitude and direction, as well as a non‐significant MR‐PRESSO distortion test. Nonetheless, another important potentially pleiotropic pathway that needs consideration is body size and composition, because a large proportion of included GSw SNPs are also associated with various anthropometric indicators, e.g., BMI, total and regional fat‐ and fat‐free mass [28]. It is likely that the effect of body composition operates upstream of GS [29], therefore representing correlated pleiotropy whereby body composition confounds the association between GS and CRF. However, we were encouraged to observe that the effect of GSw on CRFw remained consistent even after removing all SNPs associated with body size (and other confounders), although CIs then straddled the null. The second scenario by which a person's genetic predisposition to higher GSw may increase their CRFw is via a causal pathway, although underlying pathways warrant exploration. Third, as explained above, higher GSw may be linked to improved CRFw as a result of the behaviors that improve strength.

It is important to note that our GSw instrument was obtained from a GWAS performed on grip strength standardized by body weight [17] and so our instrument should be interpreted as providing the effect of GS on CRF for a given body weight. There is debate about whether GS should be standardized to weight, with some arguing that standardization weakens the association between absolute grip strength and overall muscle strength [30], whilst others argue that relative GS removes the confounding effects of body size [17] and is more suitable to reflect general muscular fitness than absolute grip strength [31]. Moreover, similar debates exist regarding standardization of CRF [32]. Given the lack of consensus, we acknowledge that standardizing by weight means we are no longer considering the effect of strength per se. While further work on the most appropriate standardizing metric is warranted, some evidence suggests that GS scaled for weight is superior to absolute GS in representing metabolic aspects of sarcopenia [33]. Additionally, we are unable to disentangle the effects of fat mass and fat‐free mass, which may have resulted in different associations with CRF [32]. Ultimately, a pragmatic decision was made to standardize both GS and CRF by weight in order to provide a consistent standardization and interpretation across both MR analyses.

A major strength of our study is that by using a bidirectional approach, we have been able to establish the direction of effect between CRFw and GSw. Within our bidirectional MR framework, we used four different methods (IVW, MR‐Egger, WME, and MR‐PRESSO) which have distinct strengths and assumptions; for example, the use of MR‐Egger and WME‐enabled analysis and minimization of pleiotropic effects. The general agreement across these different analytical models strengthens the causal interpretation of the findings. The availability of GS phenotype data in almost 400,000 UKB participants means our CRFwꟷGSw MR analysis was powered to estimate effects precisely.

We acknowledge study limitations. As CRF phenotypic data were not available in UKB, it was only possible to perform a summary‐level MR in the direction of GSw‐CRFw using the genome‐wide summary‐level statistics, based on ~80 000 UKB participants, made available for the CRFw instrument. In contrast, for the CRFw‐GSw MR analysis, we used individual‐level GS data from almost 400,000 UKB participants. Genetic variants included in our CRFw and GSw instruments were both obtained from GWAS which contained UKB participants, which may have led to an overestimation of genetic associations (‘winner's curse’ [34]). While methods have been developed to examine the extent of this bias in MR estimates [34], we did not have access to the necessary CRF phenotypic data to investigate this. However, practically, the impact of such bias on MR estimates is likely small [35]. Recently, another CRF GWAS has been published using UKB participants [36], offering a potential alternative to the CRF instrument used. At the time of writing however, summary statistics from the new GWAS were not publicly available. Thus, we were unable to use the new GWAS to extract the required beta estimates of the effect of the GS SNPs on CRF. While we undertook a series of sensitivity analyses to ensure our results were robust to confounding, we acknowledge that confounding (e.g., by assortative mating) might be present. Another consideration is the use of GS as our marker of muscle strength. It is worth reflecting on GS specifically, as well as its scaling by body weight. While GS is a convenient and commonly used proxy for overall body strength, GS measures upper limb strength. Despite this, it has been shown to be a valid proxy for total limb strength [37], but, evidence regarding its utility as a proxy for overall muscle strength is equivocal [38]. As our instrument is for GSw, we are not considering strength per se, with evidence suggesting that GSw may be superior to GS [33]. Nonetheless, we acknowledge that the best scaling metric for strength (and V0_2_max) is yet to be established and could vary by age, sex, and over generations. Pragmatically, we were limited to using data available from GWAS, which also meant we were unable to stratify analyses by age or sex. Importantly, relationships between strength and CRF may be location‐specific, and we may have observed different results between CRF and strength if a measure of lower‐limb strength had been used. Finally, selection bias into UKB is evident [39], which may have been exacerbated in the CRF‐testing sub‐sample. This has to the potential to induce collider bias and bias estimates from MR analyses [40].

In conclusion, our bidirectional MR study provides novel evidence regarding the effects of CRF on GS and vice versa. We observed no evidence in support of causal links in the direction of CRF to GS. In the reverse direction, we observed a consistent and robust effect of GS on CRF, such that evidence supports causal links from greater GS to higher CRF. These findings need replication in general population samples where the impact of relevant types of physical activity on CRF and strength are examined in, for example, randomized trials. While acknowledging that different types of physical activity can improve both CRF and strength via independent mechanisms, our finding of a dominant direction of association from greater GS to higher CRF is relevant when considering how to promote physical activity guidelines. For example, while both aerobic and strength‐building activities are undoubtedly important for health, placing too much emphasis on improving/maintaining CRF is unlikely to result in maximum benefits for other fundamental components of physical fitness, particularly muscle strength. Thus, we add to the evidence base [15] to redress the balance in public health messaging, to continue highlighting the need for strength training as a complementary component to physical activities that primarily improve CRF.

### 4.1 Perspective

Physical activity guidelines recommend participating in both aerobic and strength‐based activities, targeting cardiorespiratory fitness (CRF) and muscle strength, respectively. While CRF and muscle strength are distinct phenotypes, conferring independent health benefits, they are assumed to be inter‐related, but limited evidence exists. We observed that on average over a lifetime, higher grip strength (GS) resulted in higher CRF. In the reverse direction, we observed no causal effect of CRF on GS. Our findings are relevant when considering how to promote physical activity guidelines, demonstrating that placing too much emphasis on improving/maintaining CRF is unlikely to result in maximum benefits for muscle strength.

## Conflicts of Interest

The authors declare no conflicts of interest.

## Supporting information


**Appendix S1.** Supporting Information.

## Data Availability

Data may be obtained from a third party upon approval and payment. UK Biobank data could be obtained on application from https://www.ukbiobank.ac.uk/enable‐your‐research/apply‐for‐access.
