# Supplementary material for: Unpicking Causal Relationships Between Grip Strength and Cardiorespiratory Fitness: A Bidirectional Mendelian Randomization Study
Source: Scand J Med Sci Sports. 2024 Dec 6;34(12):e14775. doi: 10.1111/sms.14775 (PMC11623293; doi:10.1111/sms.14775)
Supplement: Supplementary file 1 — Appendix S1. Supporting Information. [file SMS-34-e14775-s001.docx]

**Supplementary material: Unpicking causal relationships between grip strength and cardio-respiratory fitness. A bidirectional Mendelian randomisation study.**

**Norris T., Cooper, R., Garfield, V., Hamer, M. & Pinto Pereira S.M.**

Contents

[**Participant selection** 2](#_Toc182318616)

[**Variable ascertainment** 2](#_Toc182318617)

[**Covariates** 2](#_Toc182318618)

[**Genotyping, Imputation, quality control and linkage disequilibrium (LD)** 3](#_Toc182318619)

[**Mendelian randomization (MR) analysis methods and assumptions** 3](#_Toc182318620)

[**(i)** **Inverse-variance weighted** 4](#_Toc182318621)

[**(ii)** **MR-Egger regression** 4](#_Toc182318622)

[**(iii)** **Weighted median estimator** 5](#_Toc182318623)

[**Supplementary Table 1: STROBE-MR checklist of recommended items to address in reports of Mendelian randomization studies**^1^ ^2^ 7](#_Toc182318624)

[**Supplementary Table 2: Summary statistics describing SNP-X associations for the CRFw and GSw instruments** 22](#_Toc182318625)

[**Supplementary table 3: Removing SNPs associated with potential confounders** 25](#_Toc182318626)

[**Supplementary table 4: Removing potentially pleiotropic SNPs** 26](#_Toc182318627)

[**Supplementary table 5: Removing SNPs associated with confounders & potentially pleiotropic SNPs** 27](#_Toc182318628)

[**Supplementary figure 1: MR estimates of the bidirectional associations between CRF-GS** 28](#_Toc182318629)

[**References** 29](#_Toc182318630)

**Participant selection**

We used UKB data to (i) obtain SNP-Y_GSw_ associations when examining the direction of effect from CRF to GS and (ii) examine associations between SNPs and potential confounders. At the time of our study, UK Biobank had genetic data available for 487,409 participants. We applied individual-level quality control (QC) to exclude participants with excessive / minimal heterozygosity, sex mismatch, excessive genetic relatedness (more than 10 putative third-degree relatives in the kinship table), no consent, non-European ancestry, and missing QC metrics, leaving 408,480 participants for analysis. We further excluded participants with no GSw or confounder data (N = 40,787), leaving 367,693 participants in our analytical sample.

**Variable ascertainment**

**Covariates**

Potential confounders were identified from a directed acyclic graph constructed using the online tool DAGitty (<https://www.dagitty.net>). Information on participants’ age, sex, smoking status, alcohol intake frequency and physical activity level were collected using a self-reported questionnaire at baseline. Weight was measured using a Tanita BC-418 MA body composition analyser; height measured with a Seca-202 height measure. Townsend deprivation index was assigned based on postcode as a continuous measure, where a higher index indicates more deprivation. Smoking status was a dichotomous variable defined as not currently smoking vs. currently smoking. Alcohol intake frequency was based on the question *‘how often do you drink’*, from which a dichotomous variable was derived representing: alcohol intake less than daily vs. alcohol intake daily/almost daily. Physical activity was based on responses to the question *‘Number of days per week of vigorous physical activity lasting at least 10 minutes’*, from which a dichotomous variable was derived: active (at least 4 days/week vigorous physical activity lasting at least 10 minutes) vs. inactive (less than 4 days/week vigorous physical activity lasting at least 10 minutes). Comorbidities (arthritis, asthma and depression) were identified as binary variables from baseline self-reported illness data using UK Biobank code lists.

**Genotyping, Imputation, quality control and linkage disequilibrium (LD)**

Genotyping in UK Biobank was performed using two customised genome-wide arrays, with a genome-wide imputation performed using combined reference panels from UK10K, 1000 Genomes phase 3, and Haplotype Reference Consortium (HRC), resulting in 93,095,623 autosomal variants(1). We further applied quality control to exclude variants with the following criteria: Fisher information <0.3, missing call rate ≥ 5%, or MAF outside of 0.01–1 range.

We used linkage disequilibrium (LD) clumping to identify the most likely causal SNP at a particular locus to ensure independence of instruments. We consulted musculoskeletal specific(2) and general(3) Mendelian Randomization study guidelines for suggested thresholds. Neither suggested the thresholds are fixed: one used *r* ^2^≤0.001 as an example(2) and the other stated that clumping cut-offs should be stated(3). Therefore, for all genetic CRF and GS variants, we used LD clumping in PLINK1.9 to ensure that SNPs were independent (thresholds: r^2^≤0.001, 250kb, p<0.01), reference haplotype data originated from the publicly released Phase 3 data from the 1000 Genomes Project(4).

**Mendelian randomization (MR) analysis methods and assumptions**

MR analysis uses genetic variants as proxies for exposure of interest, to estimate the causal association between the exposure and outcome of interest in an instrumental variable (IV) framework. A conventional MR makes assumptions that genetic instruments for the exposure must be:

- Robustly associated with the exposure. This assumption is reasonable as the genetic variants for each of the traits used in our study achieved genome-wide significance in their respective (large-scale) GWAS.
- Associated with the outcome only through exposure.
- Independent of confounders that influence the exposure and outcome after conditioning on observed confounders (e.g., area-level deprivation, smoking status, physical activity, age at recruitment, alcohol intake and comorbidities)(5).

To check the last two assumptions we (i) used MR-Egger and MR-PRESSO and (ii) regressed CRFw and GSw SNPs on potential confounders listed above using linear or logistic regression (as appropriate). We applied a Benjamini-Hochberg false discovery rate (BH-FDR) of 0.05 to account for multiple testing. We did not examine associations between SNPs and potential confounders via Phenoscanner because individual level data was available in UK Biobank. Examining these associations using the latter (when available) is optimal because the former may be based on different methodologies (e.g., quality control practices) and populations.

In this study, we utilised a bidirectional multi-instrument, two-sample (and *pseudo two-sample*) MR approach (where summary statistics for genetic associations with the exposure and the outcome were used to calculate the MR estimate). Specifically, we used the following four MR methods:

1. **Inverse-variance weighted**

For uncorrelated genetic variant $j$, the causal estimate $\hat{\beta}_{IVW}$ can be calculated by averaging the ratio between variant-outcome association estimate $\hat{\beta}_{Y_{j}}$ and variant-exposure association estimate $\hat{\beta}_{X_{j}}$ using the inverse-variance weighted formula for a multiplicative random-effect meta-analysis model, which yields:

$$\hat{\beta}_{IVW}= \frac{\sum_{j} \hat{\beta}_{Y_{j}}\hat{\beta}_{X_{j}}\sigma_{Y_{j}}^{-2}}{\sum_{j} \hat{\beta}_{X_{j}}^{2}\sigma_{Y_{j}}^{-2}}$$

where $\hat{\beta}_{Y_{j}}$ (or $\hat{\beta}_{X_{j}}$) and $\sigma_{Y_{j}}$ are the coefficient and standard error from regression of, e.g., genetic variant $j$ on GSw ($Y)$ and genetic variant $j$ on CRFw ($X)$.(6)

1. **MR-Egger regression**

The MR-Egger regression(7) performs a weighted linear regression of $\hat{\beta}_{Y_{j}}$ on the $\hat{\beta}_{X_{j}}$, using the $\sigma_{Y_{j}}^{-2}$ as weights and with unconstrained intercept^11^. The estimate $\hat{\beta}_{E}$ is obtained from the model:

$$\hat{\beta}_{Y_{j}}= \hat{\alpha}_{E}+\hat{\beta}_{E} \hat{\beta}_{X_{j}}$$

The intercept term $\hat{\alpha}_{E}$ denotes the estimated horizontal pleiotropic effect across genetic variants, and thus, the associated *p*-value is indicative of overall horizontal pleiotropy. The MR-Egger estimates typically have low power, but we used this method primarily to detect horizontal pleiotropy(8).

Both the IVW and MR-Egger models make several additional assumptions, including NO Measurement Error (NOME), InSIDE (Instrument Strength Independent of Direct Effect), and VIS (Variation in Instrument Strength), as detailed elsewhere(9).

1. **Weighted median estimator**

In the weighted median estimator, first the causal estimate $\hat{\beta}$ of each variant $j$ is calculated with the ratio method as $\hat{\beta}_{j}={\hat{\beta}_{Y_{j}}}/{\hat{\beta}_{X_{j}}}$. Then, $\hat{\beta}_{j}$ are sorted (so that $\hat{\beta}_{1}< \hat{\beta}_{2}<\ldots< \hat{\beta}_{J}$) and standardised weight $w_{j}$ is assigned to the $j$th-ordered ratio estimate. The weights are calculated using the inverse variance of the ratio estimates as $\acute{w}_{j}=\hat{\beta}_{X_{j}}\sigma_{Y_{j}}^{-2}$. The standardised weights are $w_{j}={\acute{w}_{j}}/{\sum_{j} \acute{w}_{j}}$ and their sum is $s_{j}= 1$.

If $k$ denotes the largest integer such that the sum of weights up to and including the $k$th estimate ($s_{k}= \sum_{j\leq k} w_{j}$ ) is <0.5, the causal estimate from weighted median method $\hat{\beta}_{WME}$ can be calculated by interpolation between the $k$th and $(k+1)$th ratio estimates as follows:

$$\hat{\beta}_{WME}= \hat{\beta}_{k}+\left( \hat{\beta}_{k+1}- \hat{\beta}_{k} \right) \times\frac{0.5- s_{k}}{s_{k+1}- s_{k}}$$

This approach should provide a consistent estimate given that at least 50% of the weights are derived from valid variants and is more robust to violation of the untestable InSIDE assumption(10, 11).

**(iv) MR-PRESSO**

MR-PRESSO (Mendelian Randomization Pleiotropy RESidual Sum and Outlier) allows for the evaluation of pleiotropy in a standard MR model. MR-PRESSO has three components, including: 1) detection of pleiotropy (MR-PRESSO global test); 2) correction of pleiotropy via outlier removal (MR-PRESSO outlier test); and 3) testing of significant distortion in the causal estimate before and after MR-PRESSO correction (MR-PRESSO distortion test). Further details can be found here(12).

# **Supplementary Table 1: STROBE-MR checklist of recommended items to address in reports of Mendelian randomization studies**^1^ ^2^

| **Item No.** | **Section** | **Checklist item** | **Page No.** | **Relevant text from manuscript** |
| --- | --- | --- | --- | --- |
| 1 | **TITLE and ABSTRACT** | Indicate Mendelian randomization (MR) as the study’s design in the title and/or the abstract if that is a main purpose of the study | 1 & 2 | Page 1 “Unpicking causal relationships between grip strength and cardio-respiratory fitness. A bidirectional Mendelian randomisation study.”  Page 2 “Objectives: … We performed a Mendelian Randomization (MR) analysis to elucidate the bidirectional relationship between CRF and grip strength (GS).” |
|  | **INTRODUCTION** |  |  |  |
| 2 | **Background** | Explain the scientific background and rationale for the reported study. What is the exposure? Is a potential causal relationship between exposure and outcome plausible? Justify why MR is a helpful method to address the study question | 3/4 | See “Introduction” |
| 3 | **Objectives** | State specific objectives clearly, including pre-specified causal hypotheses (if any). State that MR is a method that, under specific assumptions, intends to estimate causal effects | 3/4 and 6/7 | Page 3/4: “Thus, to address current knowledge gaps, we aim to identify whether, on average over a lifetime, a prevailing direction of causality exists between CRF and muscle strength. We do this using genetic instruments for CRF and muscle strength obtained from published genome wide association studies (GWAS)(17, 18) in combination with phenotypic data from participants in the UK Biobank study (UKB)(19). Specifically, the instruments used were for estimated V02-max (from a submaximal test) and grip strength (GS; a commonly used proxy for overall muscle strength); both were scaled for body weight.”  Page 6/7: “For MR analyses to be valid, three key assumptions must be met: i) genetic variants should be robustly associated with the exposure; ii) genetic variants should be independent of confounding factors of the relationship in question; and iii) the association between the genetic variants for the exposure and the outcome must only operate via the exposure under study (i.e., no unbalanced horizontal pleiotropy).” |
|  | **METHODS** |  |  |  |
| 4 | **Study design and data sources** | Present key elements of the study design early in the article. Consider including a table listing sources of data for all phases of the study. For each data source contributing to the analysis, describe the following: | 5, Table 1, Figure 1 and Supplementary methods | Page 5: “Study design: We employed a bidirectional Mendelian randomisation (MR) approach, whereby summary-level genome-wide association data were combined with individual-level phenotypic data (Figure 1, Table 1). Specifically, in the direction of GS (exposure) to CRF (outcome) we used summary-level data for both GS and CRF, obtained from their respective GWAS(17, 18). In the direction of CRF (exposure) to GS (outcome), we combined summary-level data for CRF with individual-level genetic & phenotypic data obtained from 367,693 UKB participants. Below we describe the phenotypic measures used from UKB and our adopted MR approaches; for more details see ‘Supplementary methods’.” |
|  | a) | Setting: Describe the study design and the underlying population, if possible. Describe the setting, locations, and relevant dates, including periods of recruitment, exposure, follow-up, and data collection, when available. | 5 | (In addition to immediately previous text):  “Phenotypic measures in UKB: grip strength and weight measurement  UKB is a large prospective cohort of individuals aged 40–69 years at recruitment (2006-2010) from across the UK(19). The sample examined here included 367,693 European ancestry participants with available data on genotypes and, at baseline, valid weight and GS measures (details in Supplementary methods). Grip strength was assessed using a Jamar J00105 hydraulic hand dynamometer. Participants sat upright in a chair with their forearms on armrests. They were asked to squeeze the dynamometer’s handle as hard as they could with their right hand for about 3 seconds. The grip strength measurement was then repeated using the same protocol for the left hand(21). We examine the maximum recorded value (greater than 0) from either hand. Weight (kg) was measured using a Tanita BC-418 MA body composition analyser. To be consistent with the scaling metric of genetic instruments for GS and CRF(17, 18), we divided GS by weight to obtain relative grip strength, i.e., grip strength per kg of body weight (GSw).” |
|  | b) | Participants: Give the eligibility criteria, and the sources and methods of selection of participants. Report the sample size, and whether any power or sample size calculations were carried out prior to the main analysis | Figure 1 | See Figure 1 |
|  | c) | Describe measurement, quality control and selection of genetic variants | 5/6 and Supplementary methods | Page 5/6: “GS to CRF (two-sample MR with overlapping samples): Obtaining SNP-X[GS] and SNP-Y[CRF] coefficients  We use summary level data from a GWAS on GS/weight(17). Hence, our instrument is for GS standardised by weight (GSw). This instrument (SNP-XGSw) comprised 109 near-independent SNPs which achieved genome-wide significance (p<5x10-8); details in Supplementary methods, Figure 1 and Table 1. These 109 SNPs were then extracted from the CRF GWAS(18) (SNP-YCRFw), which was also standardised by weight (details below). Where necessary, SNP-XGSw beta coefficients were multiplied by -1 to ensure all betas represented an increase in GSw. Next, allele harmonisation was done, ensuring alignment of SNP-YCRFw alleles to SNP-XGSw associations. The 109-SNP instrument for SNP-XGSw had an F-statistic of 34.38, explaining 1.01% of variability in GSw.  CRF to GS (pseudo two-sample MR): Obtaining SNP-X[CRF] and SNP-Y[GS] coefficients  We leveraged data from a CRF (scaled for weight) GWAS for estimated V02-max (from submaximal testing)(18). We curated an instrument (SNP-XCRFw) comprising 11 near-independent SNPs which achieved genome-wide significance; details in Supplementary methods, Figure 1 and Table 1. F-statistic and variance explained details were not reported in the original GWAS(18). Additionally, as CRF phenotypic data was not available in UKB, it was not possible to calculate the CRF instrument’s F-statistic or proportion of CRF variance explained using individual-level data. Thus, we calculated an approximate F-statistic using the ‘t-statistic’ method(20); this value was 37.72. To obtain SNP-YGSw associations, these 11 SNPs were regressed against GSw in 367,693 UKB participants (further details in Supplementary methods).” |
|  | d) | For each exposure, outcome, and other relevant variables, describe methods of assessment and diagnostic criteria for diseases | 5/6 and Supplementary methods | See previous text above. |
|  | e) | Provide details of ethics committee approval and participant informed consent, if relevant | 7 | Ethics statement  Ethical approval for UKB was given by the National Information Governance Board for Health and Social Care and North-West Multicentre Research Ethics Committee. The approval covered the analysis of all data in the present study. Participants provided informed consent; the current study is approved by UKB (application number:71702). |
| 5 | **Assumptions** | Explicitly state the three core IV assumptions for the main analysis (relevance, independence and exclusion restriction) as well assumptions for any additional or sensitivity analysis | 6/7 and Supplementary methods | Page 6/7: “For MR analyses to be valid, three key assumptions must be met: i) genetic variants should be robustly associated with the exposure; ii) genetic variants should be independent of confounding factors of the relationship in question; and iii) the association between the genetic variants for the exposure and the outcome must only operate via the exposure under study (i.e., no unbalanced horizontal pleiotropy). We explored the validity of these assumptions by testing associations between SNPs and potential confounders (sex, height, weight, depression, arthritis, asthma, area-level deprivation, smoking status, physical activity, age and alcohol intake) in UKB. We applied a Benjamini-Hochberg false discovery rate of 0.05 to account for multiple testing. Where associations were observed, MR analyses were re-run excluding potentially invalid SNPs. Additionally, when the MR-Egger intercept indicated pleiotropy (p<0.05), we undertook further analyses: outlying SNPs and those with a large influence on the estimates were identified by (i) funnel plots and (ii) Cook’s Distance(22). We reran analyses removing the identified SNPs (details in Supplementary methods).” |
| 6 | **Statistical methods: main analysis** | Describe statistical methods and statistics used | 6 and Supplementary methods | Page 6 “The described analyses were performed initially with GSw instruments as the exposure and CRFw as the outcome and then vice-versa.  We applied the inverse-variance weighted (IVW) method as our main MR model. This method estimates the causal effect of an exposure on an outcome by averaging the genetic instruments’ ratio of instrument–outcome (SNP-Y) to instrument–exposure (SNP-X) association estimates using a multiplicative random effects meta-analysis model. We report the I2 statistic to quantify the extent of heterogeneity between SNP-specific causal estimates. For the only individual level genetic association estimated (i.e., SNP-YGSw) via linear regression in PLINK 2.0, we adjusted for 10 genetic principal components. We performed three additional MR analyses: MR-Egger, weighted median estimator (WME) and MR-PRESSO. MR-Egger yields an intercept term which indicates the presence of unbalanced horizontal pleiotropy (i.e., if genetic instruments are associated with the outcome via pathways other than the exposure); WME provides more robust estimates when up to 50% of the genetic variants are invalid and MR-PRESSO identifies and corrects for potential outliers (details in Supplementary methods).” |
|  | a) | Describe how quantitative variables were handled in the analyses (i.e., scale, units, model) | 4 & 5 | Page 4: “Specifically, the instruments used were for estimated V02-max (from a submaximal test) and grip strength (GS; a commonly used proxy for overall muscle strength); both were scaled for body weight.”  Page 5: “To be consistent with the scaling metric of genetic instruments for GS and CRF(17, 18), we divided GS by weight to obtain relative grip strength, i.e., grip strength per kg of body weight (GSw).” |
|  | b) | Describe how genetic variants were handled in the analyses and, if applicable, how their weights were selected | 5/6 and Supplementary methods | See text in response to 4c |
|  | c) | Describe the MR estimator (e.g. two-stage least squares, Wald ratio) and related statistics. Detail the included covariates and, in case of two-sample MR, whether the same covariate set was used for adjustment in the two samples | 6 and Supplementary methods | See text in response to 6. |
|  | d) | Explain how missing data were addressed | NA | No missing data (explicitly stated in Supplementary methods) |
|  | e) | If applicable, indicate how multiple testing was addressed | 7 | “We applied a Benjamini-Hochberg false discovery rate of 0.05 to account for multiple testing” |
| 7 | **Assessment of assumptions** | Describe any methods or prior knowledge used to assess the assumptions or justify their validity | 6/7, Supplementary methods and supplementary tables 3-5 | See text in response to 5 and supplementary tables 3-5 |
| 8 | **Sensitivity analyses and additional analyses** | Describe any sensitivity analyses or additional analyses performed (e.g. comparison of effect estimates from different approaches, independent replication, bias analytic techniques, validation of instruments, simulations) | 6/7 and supplementary tables 3-5 | See text in response to 5 and supplementary tables 3-5 |
| 9 | **Software and pre-registration** |  |  |  |
|  | a) | Name statistical software and package(s), including version and settings used | 7 | “We used Stata17, R4.3.0 and PLINK1.9/2.0 for data processing and statistical analyses. MR analyses were performed using the mrrobust (Stata)(23) and MR-PRESSO (R)(24) packages.” |
|  | b) | State whether the study protocol and details were pre-registered (as well as when and where) | NA | NA |
|  | **RESULTS** |  |  |  |
| 10 | **Descriptive data** |  |  |  |
|  | a) | Report the numbers of individuals at each stage of included studies and reasons for exclusion. Consider use of a flow diagram | Figure 1 and Supplementary methods | Supplementary methods “Participant selection  We used UKB data to (i) obtain SNP-YGSw associations when examining the direction of effect from CRF to GS and (ii) examine associations between SNPs and potential confounders. At the time of our study, UK Biobank had genetic data available for 487,409 participants. We applied individual-level quality control (QC) to exclude participants with excessive / minimal heterozygosity, sex mismatch, excessive genetic relatedness (more than 10 putative third-degree relatives in the kinship table), no consent, non-European ancestry, and missing QC metrics, leaving 408,480 participants for analysis. We further excluded participants with no GSw or confounder data (N = 40,787), leaving 367,693 participants in our analytical sample.” |
|  | b) | Report summary statistics for phenotypic exposure(s), outcome(s), and other relevant variables (e.g. means, SDs, proportions) | Table 2 | Table 2 |
|  | c) | If the data sources include meta-analyses of previous studies, provide the assessments of heterogeneity across these studies | NA | NA |
|  | d) | For two-sample MR:  i.  Provide justification of the similarity of the genetic variant-exposure associations between the exposure and outcome samples  ii.  Provide information on the number of individuals who overlap between the exposure and outcome studies | 12 and Table 1 | Page 12: “Genetic variants included in our CRFw and GSw instruments were both obtained from GWAS which contained UKB participants, which may have led to an overestimation of genetic associations (‘winner’s curse’(34)). While methods have been developed to examine the extent of this bias in MR estimates(34), we did not have access to the necessary phenotypic data (e.g., CRF phenotypic data) required to investigate this. However, practically, the impact of such bias on MR estimates is likely small(35).” |
| 11 | **Main results** |  |  |  |
|  | a) | Report the associations between genetic variant and exposure, and between genetic variant and outcome, preferably on an interpretable scale | Supplementary Table 2 | Supplementary Table 2 |
|  | b) | Report MR estimates of the relationship between exposure and outcome, and the measures of uncertainty from the MR analysis, on an interpretable scale, such as odds ratio or relative risk per SD difference | Table 3; supplementary figure 1 | Table 3; supplementary figure 1 (and described in word in the Results section) |
|  | c) | If relevant, consider translating estimates of relative risk into absolute risk for a meaningful time period | NA | NA |
|  | d) | Consider plots to visualize results (e.g. forest plot, scatterplot of associations between genetic variants and outcome versus between genetic variants and exposure) | Supplementary Figure 1 | Supplementary Figure 1 |
| 12 | **Assessment of assumptions** |  |  |  |
|  | a) | Report the assessment of the validity of the assumptions | 8/9, table 3 and supplementary tables 3-5 | Page 8/9: “Many SNPs (102) included in the GSw instrument were associated with examined potential confounders. When these SNPs were removed and analysis re-run, effects were consistent with those in the main analysis in terms of direction and magnitude, but confidence intervals straddled the null (supplementary table 3). For example, the IVW estimate suggested a 1-unit increase in GSw was associated with a 1.66ml/kg/min (95% confidence interval (CI): -0.14, 3.47) higher CRFw. While MR-Egger did not provide evidence of an overall effect of horizontal pleiotropy (MREgger p-valueintercept=0.27), funnel plots and Cook’s Distance identified seven potentially pleiotropic SNPs (rs115131074, rs117642368, rs13135092, rs17024393, rs17249398, rs181617194, rs76808502). When analysis was re-run without these SNPs, consistent effects to those reported above were observed. For example, the IVW estimate suggested a 1-unit increase in GSw was associated with a 1.16ml/kg/min (95% CI:1.10, 2.22) higher CRFw (supplementary table 4). Re-running analyses after removing all 103 SNPs associated with confounders and/or showing potentially pleiotropic effects resulted in directionally consistent estimates, but with confidence intervals straddling the null (e.g. IVW effect: 1.18ml/kg/min (95% CI:-0.83, 3.19) higher CRFw) (supplementary table 5). Similarly, MR-PRESSO provided consistent results. The global test was significant (p<0.001), indicating the presence of some horizontal pleiotropy. However, this only had a small effect on the causal estimate, as indicated by a non-significant MR-PRESSO distortion test (p=0.71) and an outlier-adjusted estimate of 1.63ml/kg/min (Table 3).”  And  “Removing SNPs associated with confounders and/or showing potentially pleiotropic effects did not substantively change estimates (supplementary tables 3-5). MR-PRESSO indicated the presence of some horizontal pleiotropy (global test p<0.001), but this had no substantial impact on the causal effect (or lack thereof) of CRFw on GSw (Table 3).” |
|  | b) | Report any additional statistics (e.g., assessments of heterogeneity across genetic variants, such as *I^2^*, Q statistic or E-value) | 8/9, table 3 and supplementary tables 3-5 | See text in response to 12 (a). |
| 13 | **Sensitivity analyses and additional analyses** |  |  |  |
|  | a) | Report any sensitivity analyses to assess the robustness of the main results to violations of the assumptions | 8/9, table 3 and supplementary tables 3-5 | See text in response to 12 (a). |
|  | b) | Report results from other sensitivity analyses or additional analyses | 8/9, table 3 and supplementary tables 3-5 | See text in response to 12 (a). |
|  | c) | Report any assessment of direction of causal relationship (e.g., bidirectional MR) | Table 3 | (See entire manuscript on this point) |
|  | d) | When relevant, report and compare with estimates from non-MR analyses | NA | NA |
|  | e) | Consider additional plots to visualize results (e.g., leave-one-out analyses) | NA | NA |
|  | **DISCUSSION** |  |  |  |
| 14 | **Key results** | Summarize key results with reference to study objectives | 10 | Page 10 “We investigated evidence for causal links between CRF and GS in both directions in large-scale GWAS and UKB, using several complementary MR approaches and found important differences in terms of the postulated directions of association. In the direction CRF-GS, all MR estimates were consistent with the null, suggesting, on average over a lifetime, no causal effect of CRF on GS. In the reverse direction, estimates of the effect GS on CRF were directionally consistent across all MR analyses, indicating, on average over a lifetime, higher GS resulted in higher CRF.” |
| 15 | **Limitations** | Discuss limitations of the study, taking into account the validity of the IV assumptions, other sources of potential bias, and imprecision. Discuss both direction and magnitude of any potential bias and any efforts to address them | 12 | Page 12 “We acknowledge study limitations. As CRF phenotypic data were not available in UKB, it was only possible to perform a summary-level MR in the direction of GSw-CRFw using the genome wide summary level statistics, based on ~80,000 UKB participants, made available for the CRFw instrument. In contrast, for the CRFw-GSw MR analysis, we used individual level GS data from almost 400,000 UKB participants. Genetic variants included in our CRFw and GSw instruments were both obtained from GWAS which contained UKB participants, which may have led to an overestimation of genetic associations (‘winner’s curse’(34)). While methods have been developed to examine the extent of this bias in MR estimates(34), we did not have access to the necessary CRF phenotypic data to investigate this. However, practically, the impact of such bias on MR estimates is likely small(35). Recently, another CRF GWAS has been published using UKB participants(36), offering a potential alternative to the CRF instrument used. At the time of writing however, summary statistics from the new GWAS were not publicly available. Thus, we were unable to use the new GWAS to extract the required beta estimates of the effect of the GS SNPs on CRF. While we undertook a series of sensitivity analyses to ensure our results were robust to confounding, we acknowledge that confounding (e.g., by assortative mating) might be present. Another consideration is the use of GS as our marker of muscle strength. It is worth reflecting on GS specifically, as well as its scaling by body weight. While GS is a convenient and commonly used proxy for overall body strength, GS measures upper limb strength. Despite this, it has been shown to be a valid proxy for total limb strength(37), but, evidence regarding its utility as a proxy for overall muscle strength is equivocal(38). As our instrument is for GSw, we are not considering strength per se, with evidence suggesting that GSw may be superior to GS(33). Nonetheless we acknowledge that the best scaling metric for strength (and V02max) is yet to be established and could vary by age, sex and over generations. Pragmatically, we were limited to using data available from GWAS, which also meant we were unable to stratify analyses by age or sex. Importantly, relationships between strength and CRF may be location specific, and we may have observed different results between CRF and strength if a measure of lower-limb strength had been used. Finally, selection bias into UKB is evident(39), which may have been exacerbated in the CRF-testing sub-sample. This has to the potential to induce collider bias and bias estimates from MR analyses(40).” |
| 16 | **Interpretation** |  |  |  |
|  | a) | Meaning: Give a cautious overall interpretation of results in the context of their limitations and in comparison with other studies | 13 | Page 13 “In conclusion, our bidirectional MR study provides novel evidence regarding the effects of CRF on GS and vice versa. We observed no evidence in support of causal links in the direction of CRF to GS. In the reverse direction, we observed a consistent and robust effect of GS on CRF, such that evidence supports causal links from greater GS to higher CRF. These findings need replication in general population samples where the impact of relevant types of physical activity on CRF and strength are examined in, for example, randomised trials. While acknowledging that different types of physical activity can improve both CRF and strength via independent mechanisms, our finding of a dominant direction of association from greater GS to higher CRF is relevant when considering how to promote physical activity guidelines. For example, while both aerobic and strength-building activities are undoubtedly important for health, placing too much emphasis on improving/maintaining CRF is unlikely to result in maximum benefits for other fundamental components of physical fitness, particularly muscle strength. Thus, we add to the evidence base(16) to redress the balance in public health messaging, to continue highlighting the need for strength training as a complementary component to physical activities that primarily improve CRF.” |
|  | b) | Mechanism: Discuss underlying biological mechanisms that could drive a potential causal relationship between the investigated exposure and the outcome, and whether the gene-environment equivalence assumption is reasonable. Use causal language carefully, clarifying that IV estimates may provide causal effects only under certain assumptions | 10/11 | Page 10/11 “While it is unclear how a person’s genetic predisposition to higher GS may increase their CRF, at least three situations warrant consideration. First, the role of horizontal pleiotropy, in which SNPs are associated with CRF via other traits (which are not downstream of strength), needs to be considered, as indicated by the significant MR-PRESSO global test. For example, several SNPs included in the GSw instrument (e.g., rs10807136, rs10203386, rs11645565, rs117642368, rs62004866, rs1642294, rs34633411) have exhibited relationships with forced expiratory volume(28). This indicates a potential role of the included SNPs on lung function, which can then plausibly be related to CRF levels. Indeed, SNP rs117642368 was identified as one of the SNPs demonstrating high influence on the GSwꟷCRFw MR estimate, indicating a potentially horizontal pleiotropic effect. Two further SNPs are associated with functioning of cardiac muscle tissue (rs1627854 and rs4952499), which may represent another pleiotropic pathway via which strength and CRF may be associated. Reassuringly, when analyses were re-run excluding all identified potential pleiotropic SNPs, the effect of GSw on CRFw still persisted (per unit increase in GSw, CRFw was higher by 1.16ml/kg/min (95% CI:1.10, 2.22), IVW estimate). Moreover, our MR-Egger and MR-PRESSO analyses did not indicate a substantial effect of horizontal pleiotropy as evidenced by the concordant outlier-adjusted estimates, which were similar in magnitude and direction, as well as a non-significant MR-PRESSO distortion test. Nonetheless, another important potentially pleiotropic pathway that needs consideration is body size and composition, because a large proportion of included GSw SNPs are also associated with various anthropometric indicators, e.g., BMI, total and regional fat- and fat-free mass(28). It is likely that the effect of body composition operates upstream of GS(29), therefore representing correlated pleiotropy whereby body composition confounds the association between GS and CRF. However, we were encouraged to observe that the effect of GSw on CRFw remained consistent even after removing all SNPs associated with body size (and other confounders), although CIs then straddled the null. The second scenario by which a person’s genetic predisposition to higher GSw may increase their CRFw, is via a causal pathway, although underlying pathways warrant exploration. Thirdly, as explained above, higher GSw may be linked to improved CRFw as a result of the behaviours that improve strength.” |
|  | c) | Clinical relevance: Discuss whether the results have clinical or public policy relevance, and to what extent they inform effect sizes of possible interventions | 13 | Page 13: “Perspective  Physical activity guidelines recommend participating in both aerobic and strength-based activities, targeting cardiorespiratory fitness (CRF) and muscle strength, respectively. While CRF and muscle strength are distinct phenotypes, conferring independent health benefits, they are assumed to be inter-related, but limited evidence exists. We observed that on average over a lifetime, higher grip strength (GS) resulted in higher CRF. In the reverse direction, we observed no causal effect of CRF on GS. Our findings are relevant when considering how to promote physical activity guidelines, demonstrating that placing too much emphasis on improving/maintaining CRF is unlikely to result in maximum benefits for muscle strength.” |
| 17 | **Generalizability** | Discuss the generalizability of the study results (a) to other populations, (b) across other exposure periods/timings, and (c) across other levels of exposure | 12/13 | Page 12/13 “Pragmatically, we were limited to using data available from GWAS, which also meant we were unable to stratify analyses by age or sex. Importantly, relationships between strength and CRF may be location specific, and we may have observed different results between CRF and strength if a measure of lower-limb strength had been used. Finally, selection bias into UKB is evident(39), which may have been exacerbated in the CRF-testing sub-sample. This has to the potential to induce collider bias and bias estimates from MR analyses(40).” |
|  | **OTHER INFORMATION** |  |  |  |
| 18 | **Funding** | Describe sources of funding and the role of funders in the present study and, if applicable, sources of funding for the databases and original study or studies on which the present study is based | 14 | “The work was supported by a UK Medical Research Council Career Development Award (ref: MR/P020372/1) and Senior Non-clinical fellowship (ref: MR/Y009398/1) awarded to S.M.P.P. RC acknowledges support from the National Institute for Health and Care Research (NIHR) Newcastle Biomedical Research Centre. RC also receives support as part of a generous donation made by the McArdle family to Newcastle University for research that will benefit the lives of older people in the UK. V.G. was supported by a Professor David Matthews Non-Clinical Fellowship from the Diabetes Research and Wellness Foundation (ref: SCA/01/NCF/22). MH is supported by a British Heart Foundation grant (SP/F/20/150002) and NIHR University College London Hospitals Biomedical Research Centre. The views expressed in the publication are those of the authors and not necessarily those of the funders. The funders had no input into study design; data collection, analysis, and interpretation; in the writing of the report; and in the decision to submit the article for publication. Researchers were independent of influence from study funders.” |
| 19 | **Data and data sharing** | Provide the data used to perform all analyses or report where and how the data can be accessed, and reference these sources in the article. Provide the statistical code needed to reproduce the results in the article, or report whether the code is publicly accessible and if so, where | 13 | “Data may be obtained from a third party upon approval and payment. UK Biobank data could be obtained on application from https://www.ukbiobank.ac.uk/enable-your-research/apply-for-access” |
| 20 | **Conflicts of Interest** | All authors should declare all potential conflicts of interest | 13/14 | “The authors declare no competing interests.” |

This checklist is copyrighted by the Equator Network under the Creative Commons Attribution 3.0 Unported (CC BY 3.0) license.

1. Skrivankova VW, Richmond RC, Woolf BAR, Yarmolinsky J, Davies NM, Swanson SA, et al. Strengthening the Reporting of Observational Studies in Epidemiology using Mendelian Randomization (STROBE-MR) Statement. JAMA. 2021;under review.

2. Skrivankova VW, Richmond RC, Woolf BAR, Davies NM, Swanson SA, VanderWeele TJ, et al. Strengthening the Reporting of Observational Studies in Epidemiology using Mendelian Randomisation (STROBE-MR): Explanation and Elaboration. BMJ. 2021;375:n2233.

# **Supplementary Table 2: Summary statistics describing SNP-X associations for the CRFw and GSw instruments**

| **Instrument** | **SNP ID** | **Chromosome** | **Effect allele** | **Other allele** | **Beta*** | **SE** | ***p*-value** |
| --- | --- | --- | --- | --- | --- | --- | --- |
| CRFw | rs269071 | 1 | A | G | 0.031 | 0.005 | 3.6E-10 |
| CRFw | rs10497529 | 2 | G | A | 0.085 | 0.013 | 1.0E-11 |
| CRFw | rs6801957 | 3 | T | C | 0.030 | 0.005 | 1.7E-10 |
| CRFw | rs251295 | 5 | A | G | -0.027 | 0.005 | 2.3E-08 |
| CRFw | rs111299422 | 5 | T | TA | -0.038 | 0.005 | 6.5E-14 |
| CRFw | rs58730006 | 6 | A | AT | 0.047 | 0.008 | 2.7E-09 |
| CRFw | rs11190709 | 10 | G | A | -0.046 | 0.007 | 3.8E-10 |
| CRFw | rs78291913 | 16 | C | T | 0.143 | 0.024 | 1.4E-09 |
| CRFw | rs527325496 | 17 | C | CAAA | -0.036 | 0.006 | 4.2E-09 |
| CRFw | rs139077859 | 17 | G | A | -0.033 | 0.006 | 1.7E-08 |
| CRFw | rs4811602 | 20 | G | A | -0.028 | 0.005 | 3.7E-09 |
| GSw | rs1039716 | 1 | T | C | 0.002 | 0.0003 | 6.50E-09 |
| GSw | rs11121542 | 1 | A | G | -0.003 | 0.0004 | 2.80E-10 |
| GSw | rs17024393 | 1 | T | C | 0.005 | 0.0009 | 1.50E-08 |
| GSw | rs34517439 | 1 | A | C | -0.003 | 0.0004 | 2.50E-12 |
| GSw | rs539515 | 1 | A | C | 0.003 | 0.0003 | 3.10E-21 |
| GSw | rs6428601 | 1 | A | C | 0.002 | 0.0003 | 4.30E-08 |
| GSw | rs10203386 | 2 | A | T | -0.003 | 0.0003 | 1.60E-23 |
| GSw | rs11689199 | 2 | A | G | -0.002 | 0.0003 | 5.70E-09 |
| GSw | rs13017251 | 2 | A | G | 0.002 | 0.0003 | 3.80E-08 |
| GSw | rs1627854 | 2 | A | G | 0.002 | 0.0003 | 3.50E-09 |
| GSw | rs2044469 | 2 | A | G | 0.002 | 0.0003 | 3.30E-08 |
| GSw | rs35142762 | 2 | T | C | -0.004 | 0.0004 | 5.40E-22 |
| GSw | rs3771498 | 2 | T | C | 0.002 | 0.0003 | 1.40E-14 |
| GSw | rs4952499 | 2 | T | C | -0.002 | 0.0003 | 3.40E-08 |
| GSw | rs72917544 | 2 | A | G | 0.003 | 0.0004 | 9.30E-13 |
| GSw | rs7571826 | 2 | T | C | -0.002 | 0.0003 | 3.70E-13 |
| GSw | rs76808502 | 2 | C | G | 0.005 | 0.0009 | 5.20E-09 |
| GSw | rs77012907 | 2 | A | G | 0.003 | 0.0005 | 1.20E-08 |
| GSw | rs805343 | 2 | T | C | -0.002 | 0.0003 | 1.70E-08 |
| GSw | rs1454687 | 3 | C | G | -0.002 | 0.0003 | 1.90E-10 |
| GSw | rs1513475 | 3 | T | C | -0.002 | 0.0003 | 1.50E-09 |
| GSw | rs1523766 | 3 | A | G | -0.002 | 0.0003 | 1.00E-08 |
| GSw | rs4677611 | 3 | T | C | 0.002 | 0.0003 | 3.20E-09 |
| GSw | rs4858934 | 3 | T | C | 0.002 | 0.0004 | 8.70E-09 |
| GSw | rs529200 | 3 | A | G | 0.002 | 0.0003 | 6.70E-09 |
| GSw | rs62253653 | 3 | A | G | -0.002 | 0.0003 | 9.50E-11 |
| GSw | rs73077175 | 3 | A | G | 0.002 | 0.0003 | 2.60E-08 |
| GSw | rs830643 | 3 | A | G | 0.002 | 0.0003 | 4.00E-08 |
| GSw | rs901850 | 3 | T | G | 0.002 | 0.0003 | 2.90E-08 |
| GSw | rs9877408 | 3 | A | G | 0.002 | 0.0003 | 1.40E-08 |
| GSw | rs9968060 | 3 | T | C | -0.002 | 0.0003 | 4.70E-08 |
| GSw | rs13130484 | 4 | T | C | -0.002 | 0.0003 | 1.00E-12 |
| GSw | rs13132853 | 4 | A | G | -0.002 | 0.0003 | 9.30E-09 |
| GSw | rs13135092 | 4 | A | G | 0.005 | 0.0005 | 3.20E-26 |
| GSw | rs17249398 | 4 | A | G | -0.005 | 0.0009 | 1.20E-08 |
| GSw | rs207299 | 4 | T | C | 0.002 | 0.0003 | 9.60E-09 |
| GSw | rs3113767 | 4 | T | C | 0.002 | 0.0004 | 1.30E-08 |
| GSw | rs58383713 | 4 | A | C | -0.002 | 0.0004 | 3.10E-09 |
| GSw | rs66679256 | 4 | T | C | -0.002 | 0.0003 | 9.20E-09 |
| GSw | rs7658243 | 4 | A | C | -0.002 | 0.0003 | 6.60E-09 |
| GSw | rs2126165 | 5 | A | G | -0.002 | 0.0003 | 3.80E-11 |
| GSw | rs2431112 | 5 | A | G | -0.002 | 0.0003 | 2.20E-15 |
| GSw | rs245774 | 5 | A | G | 0.002 | 0.0003 | 1.20E-11 |
| GSw | rs329120 | 5 | T | C | 0.002 | 0.0003 | 2.20E-08 |
| GSw | rs34341 | 5 | A | T | 0.002 | 0.0003 | 8.40E-10 |
| GSw | rs40067 | 5 | A | G | 0.002 | 0.0004 | 1.60E-09 |
| GSw | rs10807136 | 6 | T | C | -0.003 | 0.0004 | 3.30E-09 |
| GSw | rs12055409 | 6 | A | G | -0.002 | 0.0003 | 3.50E-08 |
| GSw | rs2237149 | 6 | A | C | 0.002 | 0.0003 | 7.50E-10 |
| GSw | rs2744475 | 6 | C | G | 0.003 | 0.0003 | 1.50E-20 |
| GSw | rs34916901 | 6 | T | G | 0.002 | 0.0003 | 3.20E-10 |
| GSw | rs6904571 | 6 | T | C | 0.002 | 0.0003 | 1.30E-08 |
| GSw | rs6905419 | 6 | T | C | -0.002 | 0.0003 | 2.40E-08 |
| GSw | rs9320823 | 6 | T | C | 0.002 | 0.0003 | 1.40E-08 |
| GSw | rs9469899 | 6 | A | G | -0.002 | 0.0003 | 2.50E-15 |
| GSw | rs10279237 | 7 | T | C | 0.002 | 0.0004 | 6.50E-09 |
| GSw | rs115131074 | 7 | A | T | -0.004 | 0.0007 | 9.00E-10 |
| GSw | rs11766468 | 7 | A | G | 0.003 | 0.0004 | 7.80E-12 |
| GSw | rs4549685 | 7 | T | C | 0.002 | 0.0003 | 4.50E-14 |
| GSw | rs62442203 | 7 | C | G | 0.002 | 0.0003 | 2.70E-09 |
| GSw | rs9641509 | 7 | T | G | 0.002 | 0.0003 | 3.10E-08 |
| GSw | rs12680855 | 8 | A | G | -0.002 | 0.0003 | 1.80E-08 |
| GSw | rs62512210 | 8 | T | C | -0.004 | 0.0005 | 1.90E-13 |
| GSw | rs10758232 | 9 | C | G | 0.002 | 0.0003 | 2.00E-08 |
| GSw | rs1329733 | 9 | A | G | 0.002 | 0.0003 | 4.30E-08 |
| GSw | rs1243182 | 10 | T | C | -0.002 | 0.0003 | 3.50E-16 |
| GSw | rs1556659 | 10 | T | C | 0.002 | 0.0003 | 1.00E-13 |
| GSw | rs563296 | 10 | A | G | -0.002 | 0.0003 | 3.40E-12 |
| GSw | rs61873510 | 10 | T | G | -0.002 | 0.0003 | 7.70E-09 |
| GSw | rs11030104 | 11 | A | G | -0.002 | 0.0003 | 8.70E-10 |
| GSw | rs11039266 | 11 | T | G | -0.003 | 0.0003 | 2.70E-18 |
| GSw | rs11223444 | 11 | A | G | -0.002 | 0.0003 | 3.20E-09 |
| GSw | rs4755720 | 11 | T | C | 0.002 | 0.0003 | 6.20E-09 |
| GSw | rs72977282 | 11 | A | T | -0.002 | 0.0003 | 9.00E-10 |
| GSw | rs12367809 | 12 | T | C | -0.002 | 0.0003 | 1.20E-11 |
| GSw | rs147730268 | 12 | T | G | 0.003 | 0.0005 | 1.80E-11 |
| GSw | rs181617194 | 12 | T | C | -0.005 | 0.0008 | 1.20E-09 |
| GSw | rs2287226 | 12 | A | G | 0.002 | 0.0003 | 1.20E-12 |
| GSw | rs4575361 | 12 | A | T | 0.002 | 0.0003 | 1.20E-10 |
| GSw | rs7955910 | 12 | T | G | -0.002 | 0.0003 | 1.40E-09 |
| GSw | rs9579775 | 13 | A | C | 0.002 | 0.0004 | 1.40E-08 |
| GSw | rs1951716 | 14 | T | C | -0.002 | 0.0003 | 2.40E-09 |
| GSw | rs202941 | 14 | T | C | 0.002 | 0.0003 | 4.20E-09 |
| GSw | rs61992671 | 14 | A | G | -0.002 | 0.0003 | 9.70E-09 |
| GSw | rs12910459 | 15 | T | C | -0.002 | 0.0003 | 3.10E-11 |
| GSw | rs1521624 | 15 | A | C | 0.002 | 0.0003 | 4.00E-08 |
| GSw | rs1529883 | 15 | C | G | 0.002 | 0.0003 | 1.30E-08 |
| GSw | rs4776614 | 15 | C | G | 0.002 | 0.0003 | 1.90E-09 |
| GSw | rs4886869 | 15 | A | G | 0.002 | 0.0003 | 2.50E-08 |
| GSw | rs62004866 | 15 | C | G | 0.003 | 0.0004 | 5.60E-10 |
| GSw | rs7165759 | 15 | A | G | 0.002 | 0.0003 | 1.30E-13 |
| GSw | rs11642015 | 16 | T | C | -0.004 | 0.0003 | 7.50E-49 |
| GSw | rs11645565 | 16 | A | G | -0.002 | 0.0003 | 7.10E-16 |
| GSw | rs12447992 | 16 | A | C | -0.002 | 0.0004 | 4.70E-09 |
| GSw | rs12599952 | 16 | A | G | 0.002 | 0.0003 | 7.30E-11 |
| GSw | rs12691307 | 16 | A | G | 0.002 | 0.0003 | 5.60E-10 |
| GSw | rs2726036 | 16 | A | C | 0.003 | 0.0003 | 3.40E-20 |
| GSw | rs62037364 | 16 | A | G | -0.003 | 0.0003 | 2.20E-26 |
| GSw | rs117642368 | 17 | C | G | -0.002 | 0.0005 | 3.40E-08 |
| GSw | rs8073510 | 17 | A | G | 0.003 | 0.0004 | 1.30E-16 |
| GSw | rs1642294 | 18 | C | G | 0.003 | 0.0004 | 5.90E-15 |
| GSw | rs303760 | 18 | T | C | -0.002 | 0.0003 | 2.00E-08 |
| GSw | rs34633411 | 18 | T | C | -0.003 | 0.0003 | 2.10E-19 |
| GSw | rs11084110 | 19 | T | C | 0.002 | 0.0003 | 1.60E-08 |
| GSw | rs113230003 | 19 | A | G | 0.002 | 0.0003 | 1.90E-11 |
| GSw | rs12162265 | 19 | A | G | -0.002 | 0.0003 | 4.60E-08 |
| GSw | rs4452060 | 19 | A | C | 0.002 | 0.0003 | 3.30E-08 |
| GSw | rs2206929 | 20 | T | C | -0.002 | 0.0003 | 1.50E-08 |
| GSw | rs5750673 | 22 | A | G | -0.002 | 0.0003 | 1.70E-08 |

*Where necessary, beta coefficients were multiplied by -1 to ensure all betas represented an increase in the respective traits and allele harmonisation was done to ensure alignment of alleles for both the SNP-X and SNP-Y associations; GWAS betas, SEs and *p*-values taken from Hanscombe et al. 2021. Genome Med, 13(1):180 (CRFw) and Tikkanen et al. 2018. Sci Rep, 8(1):6451 (GSw).

# **Supplementary table 3: Removing SNPs associated with potential confounders**

| **Difference (95% CI) in CRF (ml/kg/min)** | |
| --- | --- |
| *Number of SNPs removed* | 102 |
| IVW | 1.66 (-0.14, 3.47) |
| I^2^ | 0.0 |
| WME | 1.28 (-1.20, 3.75) |
| MR-Egger | 5.40 (-2.46, 13.27) |
| *p-*pleiotropy | 0.34 |
| **Difference (95% CI) in GS/weight (kg/kg)** | |
| *Number of SNPs removed* | 6 |
| IVW | 0.01 (-0.00, 0.02) |
| I^2^ | 0.29 |
| WME | 0.01 (-0.00, 0.02) |
| MR-Egger | 0.02 (-0.00, 0.04) |
| *p*-pleiotropy | 0.38 |

IVW: Inverse-variance-weighted; WME: weighted median estimator; MR-Egger: Mendelian randomisation Egger regression; estimates reflect associations between genetically predicted increases in CRFw on GSw and vice versa

# **Supplementary table 4: Removing potentially pleiotropic SNPs**

| **Difference (95% CI) in CRF (ml/kg/min)** | |
| --- | --- |
| *Number of SNPs removed* | 7 |
| IVW | 1.16 (1.10, 2.22) |
| I^2^ | 0.35 |
| WME | 1.70 (1.00, 2.40) |
| MR-Egger | 2.13 (-0.78, 5.03) |
| *p-*pleiotropy | 0.75 |
| **Difference (95% CI) in GS/weight (kg/kg)** | |
| *Number of SNPs removed* | 1 |
| IVW | -0.00 (-0.02, 0.01) |
| I^2^ | 0.80 |
| WME | -0.00 (-0.01, 0.01) |
| MR-Egger | 0.01 (-0.05, 0.06) |
| *p*-pleiotropy | 0.74 |

IVW: Inverse-variance-weighted; WME: weighted median estimator; MR-Egger: Mendelian randomisation Egger regression; estimates reflect associations between genetically predicted increases in CRFw on GSw and vice versa

# **Supplementary table 5: Removing SNPs associated with confounders & potentially pleiotropic SNPs**

| **Difference (95% CI) in CRF (ml/kg/min)** | |
| --- | --- |
| *Number of SNPs removed* | 103 |
| IVW | 1.18 (-0.83, 3.19) |
| I^2^ | 0.0 |
| WME | 1.23 (-1.23, 3.70) |
| MR-Egger | 1.50 (-15.76, 18.87) |
| *p-*pleiotropy | 0.97 |
| **Difference (95% CI) in GS/weight (kg/kg)** | |
| *Number of SNPs removed* | 7 |
| IVW | 0.01 (-0.00, 0.02) |
| I^2^ | 0.46 |
| WME | 0.01 (-0.00, 0.02) |
| MR-Egger | 0.03 (-0.00, 0.06) |
| *p*-pleiotropy | 0.19 |

IVW: Inverse-variance-weighted; WME: weighted median estimator; MR-Egger: Mendelian randomisation Egger regression; estimates reflect associations between genetically predicted increases in CRFw on GSw and vice versa

# **Supplementary figure 1: MR estimates of the bidirectional associations between CRF-GS**


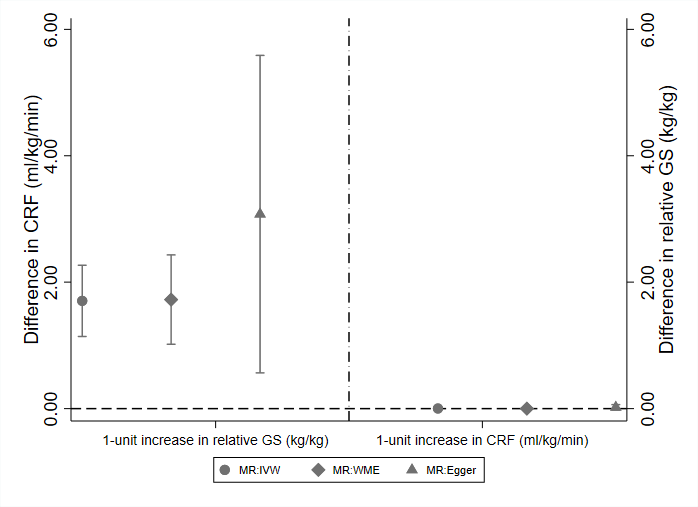


# **References**

1. Bycroft C, Freeman C, Petkova D, Band G, Elliott LT, Sharp K, et al. The UK Biobank resource with deep phenotyping and genomic data. Nature. 2018;562(7726):203-9.

2. Hartley AE, Power GM, Sanderson E, Smith GD. A guide for understanding and designing mendelian randomization studies in the musculoskeletal field. Journal of Bone and Mineral Research Plus. 2022;6(10):e10675.

3. Burgess S, Smith GD, Davies NM, Dudbridge F, Gill D, Glymour MM, et al. Guidelines for performing Mendelian randomization investigations: update for summer 2023. Wellcome open research. 2019;4.

4. Consortium G. An integrated map of genetic variation from 1,092 human genomes. Nature. 2012;491(7422):56-65.

5. Davey Smith G, Hemani G. Mendelian randomization: genetic anchors for causal inference in epidemiological studies. Human molecular genetics. 2014;23(R1):R89-R98.

6. Burgess S, Bowden J. Integrating summarized data from multiple genetic variants in Mendelian randomization: bias and coverage properties of inverse-variance weighted methods. arXiv preprint arXiv:151204486. 2015.

7. Bowden J, Davey Smith G, Burgess S. Mendelian randomization with invalid instruments: effect estimation and bias detection through Egger regression. International journal of epidemiology. 2015;44(2):512-25.

8. Burgess S, Bowden J, Fall T, Ingelsson E, Thompson SG. Sensitivity analyses for robust causal inference from Mendelian randomization analyses with multiple genetic variants. Epidemiology (Cambridge, Mass). 2017;28(1):30.

9. Bowden J, Del Greco M F, Minelli C, Davey Smith G, Sheehan N, Thompson J. A framework for the investigation of pleiotropy in two‐sample summary data Mendelian randomization. Statistics in medicine. 2017;36(11):1783-802.

10. Bowden J, Davey Smith G, Haycock PC, Burgess S. Consistent estimation in Mendelian randomization with some invalid instruments using a weighted median estimator. Genetic epidemiology. 2016;40(4):304-14.

11. Burgess S, Bowden J, Dudbridge F, Thompson SG. Robust instrumental variable methods using multiple candidate instruments with application to Mendelian randomization. arXiv preprint arXiv:160603729. 2016.

12. Verbanck M, Chen C-Y, Neale B, Do R. Detection of widespread horizontal pleiotropy in causal relationships inferred from Mendelian randomization between complex traits and diseases. Nature genetics. 2018;50(5):693-8.
